# A line through the brain: implementation of human line-scanning at 7T for ultra-high spatiotemporal resolution fMRI

**DOI:** 10.1177/0271678X211037266

**Published:** 2021-08-20

**Authors:** Luisa Raimondo, Tomas Knapen, ĺcaro A.F Oliveira, Xin Yu, Serge O Dumoulin, Wietske van der Zwaag, Jeroen C.W Siero

**Affiliations:** 1 Spinoza Centre for Neuroimaging, Amsterdam, Netherlands; 2 Experimental and Applied Psychology, VU University, Amsterdam, Netherlands; 3 MGH/MIT/HMS Athinoula A. Martinos Center for Biomedical Imaging, Department of Radiology, Harvard Medical School, Massachusetts General Hospital, Charlestown, MA, USA; 4 Experimental Psychology, 8125 Utrecht University, Utrecht University, Utrecht, Netherlands; 5 Radiology, Centre for Image Sciences, University Medical Centre Utrecht, Utrecht, Netherlands

**Keywords:** Line-scanning, high spatiotemporal resolution, fMRI, BOLD, 7T

## Abstract

Functional magnetic resonance imaging (fMRI) is a widely used tool in neuroscience to detect neurally evoked responses, e.g. the blood oxygenation level-dependent (BOLD) signal. Typically, BOLD fMRI has millimeter spatial resolution and temporal resolution of one to few seconds. To study the sub-millimeter structures and activity of the cortical gray matter, the field needs an fMRI method with high spatial and temporal resolution. Line-scanning fMRI achieves very high spatial resolution and high sampling rate, at the cost of a sacrifice in volume coverage. Here, we present a human line-scanning implementation on a 7T MRI system. First, we investigate the quality of the saturation pulses that suppress MR signal outside the line. Second, we established the best coil combination for reconstruction. Finally, we applied the line-scanning method in the occipital lobe during a visual stimulation task, showing BOLD responses along cortical depth, every 250 µm with a 200 ms repetition time (TR). We found a good correspondence of t-statistics values with 2D gradient-echo echo planar imaging (GE-EPI) BOLD fMRI data with the same temporal resolution and voxel volume (R = 0.6 ± 0.2). In summary, we demonstrate the feasibility of line-scanning in humans and this opens line-scanning fMRI for applications in cognitive and clinical neuroscience.

## Introduction

Functional magnetic resonance imaging (fMRI) is a widely used tool in neuroscience, where most fMRI studies are based on blood oxygenation level-dependent (BOLD) contrast weighted imaging.^1^ fMRI data are typically acquired using Echo Planar Imaging (EPI). EPI is an efficient sampling method, with up to submillimeter spatial resolution and a temporal resolution usually in the order of seconds. Spatially, the neurons, however, are organized in columnar and laminar structures measuring in the hundreds of micrometers.^2^ Temporally, neurons communicate at the microsecond level. The BOLD response features carry information in the range of hundreds of microseconds.^3,4^ A subsecond, preferably ∼100ms sampling rate in fMRI is necessary to detect temporal features of the hemodynamic response function (HRF) that specify how the hemodynamic signal propagates through the functional at the mesoscopic scale (cortical layers).

Advances in fMRI methodology have been aimed at increasing both the spatial and the temporal resolution of fMRI, with the final goal being sub-millimeter spatial resolution and sub-second sampling rate.

Ultra-high magnetic field strength MRI systems allow fMRI data acquisition with high spatial resolutions^5^ because of the increases in signal-to-noise ratio (SNR)^6^ and contrast-to-noise ratio (CNR)^7,8^ with the magnetic field strength B_0_. In general, different spatial resolutions are required, depending on the functional unit which are the study object. Human cortical thickness varies between 1 and 4.5 mm, with an overall average of approximately 2.5 mm.^9^ Within the cortex, different layers can be distinguished; hence sub-millimeter spatial resolution is a prerequisite in terms of spatial resolution for layer resolved fMRI studies. Here, we specifically focused on the human visual cortex which has a cortical thickness around 2 mm, and is composed of 6 layers. In this context and region, we believe that a spatial resolution of 250 µm is the minimal resolution to detect functional organization both at columnar (∼500 µm) and laminar level (∼500 µm).^2,10^ Other regions of human cortex, such as motor cortex, are slightly thicker, and contain fewer discernable layers. Here a spatial resolution of 500 µm might be adequate to resolve signals at different cortical depths, corresponding to the cortical layers.

Similar voxel sizes are required for macaque (ocular dominance columns ∼400 µm and laminae ∼100–500 µm)^11^ and other primates. However, in rodents, cortical thickness ranges from 900 µm to 3400 µm,^12^ so higher spatial resolution is required to distinguish these layers with sufficient accuracy.

Animal experiments have demonstrated cortical layer specific fMRI activations with spatial resolutions as high as ∼100–200 µm.^13–15^ Cortical depth-dependent BOLD fMRI activations in humans have also been shown in the primary visual, auditory and motor cortices, typically with spatial resolutions in the range of 750–1300 µm.^16–18^ However, an increase in spatial resolution usually comes at the cost of temporal resolution and longer scan times as more points have to be sampled to obtain the same brain coverage.

Different methods have been developed to achieve higher temporal resolutions; undersampling techniques such as partial Fourier (PF)^19^ and parallel imaging (PI),^20,21^ as well as compressed sensing^22^ are now widely available. These can also be applied to EPI sequences, but the gain in temporal resolution is not very high because of the requirement to keep the echo time (TE) close to the tissue T2*. Strongly accelerated SMS-EPI (15-fold total acceleration) sequences using a custom 32-channel coil for 7T can lead to 1.5 mm isotropic resolution with sampling rate of 1.2 s and all brain coverage.^23^

Larger speed gains can be obtained for 3D acquisition schemes, where the third dimension can be undersampled in order to obtain shorter scan time,^24,25^ similar to what has been achieved successfully with simultaneous multislice imaging.^26,27^ All these methods allow whole-brain coverage.

Other strategies need to be adopted to reach a combination of very high temporal and spatial resolution for BOLD fMRI studies, since all high-resolution EPI-based methods are still relatively slow due to the slice phase-encoding steps or multiple slice acquisitions. Acquiring only single-slice data allows a very fast acquisition, with repetition times (TR) down to ∼200 ms. In order to simultaneously increase the spatial resolution, one could reduce the in-plane field-of-view (FOV), in combination with an outer volume suppression (OVS) scheme, similar to so-called zoomed imaging.^28^ The smaller FOV allows sub-millimeter resolution and a faster sampling as a smaller imaging matrix is acquired.^29^ Specific FOVs have been adapted to map different brain regions with high in-plane spatial resolution.^30,31^ focused on ocular dominance columns in the visual cortex with elongated voxels, while Huber et al.^32^ studied the laminar activity of motor cortex with similar anisotropic voxels. Kashyap et al.^33^ achieved unprecedented 0.1 mm in-plane spatial resolution to resolve laminar activation in human visual cortex with even more anisotropic ‘pancake’ voxels, optimized for the sampling of cortical depth.

The sampling time can be further shortened if the phase-encoding steps are completely skipped. This extreme approach is dubbed line-scanning and, as the name suggests, involves the acquisition of only one line of interest. A single-slice is excited and the signal outside the line of interest can be suppressed through saturation (OVS) pulses. The phase-encoding in the direction perpendicular to the line is omitted, and the line signal is then acquired after every excitation pulse. Line-scanning fMRI has been successfully implemented by pioneering studies conducted by X. Yu et al.^34^ in rodents on a 11.7T MRI system. Line-scanning fMRI data were acquired in rodents with 50 ms temporal resolution and 50 µm spatial resolution along the line. They managed to extract high-fidelity BOLD hemodynamic response functions (HRF) of cortical laminae. Specifically, the laminar position of BOLD fMRI onsets were mapped according to the neural input applied in somatosensory and motor cortices of rats. Other preliminary studies have shown the feasibility of line-scanning in humans at 3T^35^ and promising results for neuroscience applications at 7 T.^36^ Specifically, Morgan et al. showed preliminary results identifying cortical layers in the human primary visual cortex through multi-echo line-scanning.

In this study, we investigated gradient-echo line-scanning fMRI for human applications, with in-line spatial resolution of 250 µm and temporal resolution of 200 ms. We discuss the implemented pulse sequence, data reconstruction and analysis. We compared the BOLD sensitivity of line-scanning with a fast single-slice gradient-echo BOLD EPI sequence using a block-design visual task. We previously presented initial findings on these investigations.^37,38^

## Materials and methods

### Data acquisition

Nine healthy volunteers (4 male, 30 ± 5 years old) participated in this study after providing written informed consent as approved by the medical ethics committee of the Amsterdam University Medical Centre. The guidelines of the Helsinki Declaration were followed throughout the study. All participants were screened prior to the experiments, to ensure MR compatibility.

**Figure 1.**
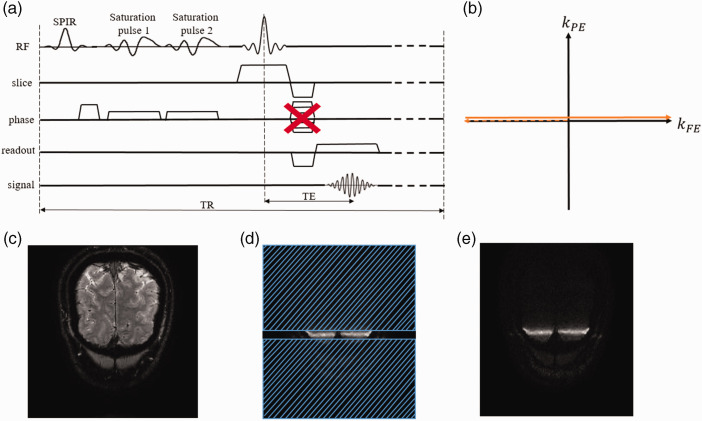
(a) Schematic of the gradient echo line-scanning (GE-line) sequence. Phase-encoding gradients are removed so that signal collapses along the phase-encoding direction into the line profile. Two saturation pulses suppress the signal outside the relevant cortical area, i.e. acting as outer volume suppression slabs. (b) The line-scanning k-space sampling pattern: acquisition of the same *k_FE_* line every TR. (c) Acquired slice. (d) outer volume suppression (OVS): placement of saturation slabs to suppress unwanted signal outside the line of interest, depicted by the gap (4 mm) between the saturation slabs, in right/left direction across visual cortex. (e) effect of the OVS on the phase-encoded slice, i.e. LSD image.

All volunteers were scanned on a 7T MRI system (Philips, Netherlands) equipped with a 2 channel transmit, 32 channel receive head coil (Nova Medical, USA). We acquired line-scanning data using a modified 2D gradient-echo (GE) sequence, depicted in Figure 1(a).

To suppress the signal outside the targeted area in the line of interest, we applied two slab-selective spatial radiofrequency (RF) saturation pulses for outer volume suppression (OVS), before slice excitation. The spatial saturation pulses had a pulse duration of 7.16 ms, a pulse flip angle of 97°, RF amplitudes of 4.85µT and 4.67 µT respectively, related gradients with 0.27 mT/m gradient strength and duration of 7.76 ms (note this includes the slope time). Fat suppression was applied before the OVS using the vendor implementation of spectral presaturation with inversion recovery (SPIR), adjusting the frequency offset to 250 Hz and bandwidth to 1000 Hz. All the prepulses (including fat suppression and OVS) were repeated every TR. The phase-encoding gradients were turned off so that the signal collapses along the phase-encoding direction into a line profile. The different ‘phase-encoding steps’ hence become time points in a functional experiment. The equivalent in k-space is the acquisition of one line, crossing the center k-space point (k_PE_ = 0), every TR (Figure 1(b)), which represents mainly the signal coming from the region between the two saturation slabs, i.e. the “line”. The parameters of the functional acquisitions were as follows: readout direction, line resolution: 250 µm, array size: 720 points along the line, line thickness in the ‘slice’ direction: 2.5 mm. The nominal in-plane line width, or gap between the two OVS slabs, was 4 mm, TR 200 ms and flip angle of 16°, with a total of 520 timepoints per run. The readout was performed with a gradient duration of 22.28 ms and strength of 4.26 mT/m, resulting in a readout bandwidth of 45.4 Hz/pixel. No SAR restrictions were encountered, vendor SAR level estimation never exceeded 47% of the local SAR limit. A TE of 13 ms was used for the first 3 subjects. In these subjects, we investigated the optimal coil combination approach, line-scanning temporal SNR and BOLD sensitivity. A TE of 22 ms was used for the other subjects, where we compared the BOLD sensitivity between the line-scanning and a fast single-slice GE-EPI sequence (see below).

In each session, one phase-encoded scan (without OVS) was acquired as an anatomical reference for the line-scanning data. Moreover, one phase-encoded scan (with OVS) was acquired before and one after the functional scans, to assess possible subject motion and to generate coil sensitivity maps. Subjects’ motion across different runs was evaluated through frame-wise displacement.^39^ Scan parameters matched that of the line as much as possible: voxel size 250 × 250 µm^2^, matrix size 720 × 180, slice thickness = 2.5 mm, TR = 103 ms, flip angle = 16° and TE = 13 ms.

The line-scanning signal is defined as the projection of all the signals originating from the slice with OVS, which we here dub the line signal distribution image (LSD image, see Figure 1(c)). Therefore, voxels can only be assigned to a certain tissue type if the signal is homogeneous along the phase-encoding direction, otherwise a mixture of tissue types is likely present due to partial volume effects. Hence, the line was, wherever possible, positioned orthogonal to the cortical ribbon across the two hemispheres, along the right-left axis and crossing the visual cortex. In Figure 1(c), an anatomical scan of the acquired slice is shown. Figure 1(b) and (c) indicate the positions of the OVS slabs and give a visual impression of their signal saturation effect outside the line (Figure 1(e)).

We acquired functional data using a block design visual task in 6 runs of 104 s each, using a strong visual stimulus to elicit robust BOLD responses in the occipital cortex. A full-field 20 Hz black and white flickering checkerboard was presented in blocks for 10 s on/off, starting with 4 s baseline and finishing with 10 s baseline. Subjects were asked to fixate on a fixation cross present during the OFF condition and in the center of the checkerboard during the ON condition.

To assess the line-scanning BOLD sensitivity, we acquired a phase-encoded fast single-slice EPI BOLD fMRI dataset in 5 volunteers for comparison. Here, fMRI data was recorded using a single-slice GE-EPI acquisition with a 1x1 mm^2^ in-plane spatial resolution and 2.5 mm slice thickness to match voxel volume in the line data. Other parameters were: matrix size 176×176, TR = 200 ms, TE = 22 ms, flip angle = 30°, SENSE factor 3, partial Fourier = 0.8. One run of 520 timepoints was acquired, leading to a total acquisition time of 104 s. Note that the temporal resolution (200 ms) was matched to the line-scanning fMRI. The same functional stimulus was used as for the line-scanning acquisitions. For a more direct comparison the value used for the line-scanning acquisition and the single-slice GE-EPI are reposted in Table 1. Notice that TR and TE were chosen to be exactly the same, as well as the overall voxel volume. Note, the FA was slightly different (16° for line-scanning and 30° for GE-EPI). However, this discrepancy leads to a small relative signal difference and should not influence the final results (less than 1.1% using the signal equation for a spoiled gradient-echo acquisition and a T_1_ of 1800 ms for gray matter tissue, see also the Discussion section).

**Table 1.** Acquisition parameters for the comparison between line-scanning and 2 D GE-EPI.

	Line-scanning	2D GE-EPI
TR	200 ms	200 ms
TE	22 ms	22 ms
FA	16°	30°
Spatial resolution	0.25 × 4×2.5 mm^3^	1 × 1×2.5 mm^3^

## Reconstruction

The reconstruction was performed offline using Matlab (Mathworks Inc, USA) and MRecon (Gyrotools, CH). Four different reconstruction methods^40^ to combine the multi-channel line-scanning data were compared.

1. Sum of squares (SoS)
2. Weighted SoS using the tSNR per coil element (tSNR)
3. Weighted SoS using the coil sensitivity maps (csm)
4. Combination of 2) and 3), as follows: $S\left(x\right)=\frac{\sum_{i}^{N_{c}}w_{i}\left(x\right)\;*\;S_{i}(x)}{\sqrt{\sum_{i}^{N_{c}}{\left|w_{i}\left(x\right)\right|}^{2}}}\;$ where *S* is the MRI signal, *Nc* is the number of channels of the receive coil (*Nc*=32), and $w_{i}\left(x\right)=\mathrm{conj}(\mathrm{csm})*\;\mathrm{tSNR}\left(x\right)\;\mathrm{per}\;\mathrm{coil}$ as the weighting factor.

Coil sensitivity maps (csm) were obtained from the phase-encoded reference scan that included OVS slabs *(Data_py_*), according to the following formula:

$$\mathrm{csm}=\;\frac{Re\left(\mathrm{Dat}a_{py}\right)+i*Im(\mathrm{Dat}a_{py})}{\sum_{i=1}^{Nc}{\left|\mathrm{Dat}a_{py}\right|}^{2}}$$

Where *Re* and *Im* indicate the real and imaginary part of *Data_py_*.

*Data_py_* were first smoothed with a 2D Gaussian smoothing kernel, with a full-width-at-half-maximum (FWHM) of 7 mm. Finally, the 2D csm were summed along the phase-encoding direction over the region where the slice was positioned, so their dimensions matched that of the functional line-scanning data. The coil combination yielding the highest tSNR of the resulting time-series was used for subsequent analyses. tSNR was evaluated through:

$$\mathrm{tSNR}=\frac{\bar{S(t)}}{\sigma (S\left(t\right))}$$

Where $\bar{S(t)}$ is the mean signal over time and $\sigma (S\left(t\right))$ is the standard deviation of the signal across time.

## Analysis

We evaluated the performance of the OVS as the ratio of the mean signal intensity between a scan with and without OVS in regions of interest (ROI) inside and outside the line. We also report the signal leakage from outside the line into the equivalent line scan area. This line-scanning signal leakage is computed as the fraction of signal from outside the line and the total signal in an equivalent line scan (i.e. complex addition of all signals in the phase-encoding direction). In the same way we evaluated the signal coming from inside the line. The mean signal profile perpendicular to the line was also computed and the FWHM was used to estimate the effective line width.

Subject motion across different runs was estimated for all sessions through the framewise displacement evaluation of the LSD images acquired in the beginning and at the end of the scan session. Additionally, the motion within single runs was evaluated through the displacement of the line center of mass (root mean square displacement). Data from one subject, presenting an average root mean squared displacement across runs higher than 0.6 mm, were excluded from the analysis.

Functional data were analyzed using a general linear model (GLM) approach to assess the line-scanning and 2D GE-EPI BOLD sensitivity. T-statistics values (t-stats) were computed to select active voxels. For the computation of line-scanning t-stats we averaged over the 6 runs, while to have a proper comparison with the GE-EPI we considered each line-scanning run separately, in order to have the same degrees of freedom (only one run of GE-EPI fMRI was acquired).

To compare line-scanning and single-slice GE-EPI fMRI acquisitions we modified the GE-EPI data in two ways, to make the 2D data comparable to line data. This double approach was chosen to confirm that the performance of the OVS was adequate for fMRI experiments and that any leaked signal from outside the line of interest did not influence the final results of the GLM analysis. First, we multiplied the LSD image by the single-slice BOLD time-series data after matching the spatial resolution of the LSD image to the one of the single slice GE-EPI (CASE1-LSD). The resulting image was summed along the phase-encoding direction to obtain “line data” for the GE-EPI, prior to computation of t-stats, giving rise to an activation profile along the line. For the second approach (CASE2-NOM), we selected the region where the line was nominally positioned in the GE-EPI scan and again summed along the phase-encoding direction in that region, before the GLM analysis. For a better understanding of the two approaches, see also Figure S1 in the supplementary material. In both cases, we manually aligned the line-scanning data to the 1D versions of the image data and averaged the line-scanning data every 4 voxels in the readout (line) direction, in order to match the spatial resolutions of the two acquisitions. Then we calculated the Spearman’s correlation (R) between the t-stats in brain tissue regions of every line-scanning run and the GE-EPI CASE1-LSD t-stats and the CASE2-NOM t-stats, yielding two correlation values (R_CASE1-LSD_ and R_CASE2-NOM_). Comparing R_CASE1-LSD_ and R_CASE2-NOM_ will allow us to quantify the out-of-line BOLD contamination. We also estimated the correlation of the t-stats between CASE1-LSD and CASE2-NOM (R_CASE1,2_). In addition, we evaluated the correlation coefficients between t-stats of every run of line-scanning with respect to each other, to estimate the stability over time. Kolmogorov-Smirnov test was performed on the distributions of t-stats for the line-scanning, CASE1-LSD and CASE2-NOM data, and it rejected the null hypothesis at the 5% significance level for all cases. For this reason, we used the non-parametric Spearman's correlation coefficient for the correlation analyses.

Finally, we report an example of cortical depth profile in a small line segment (9 voxels), as well as the temporal behavior of the same voxels. We chose a region where the line was perpendicular to the cortical surface. We averaged the signal across runs and across trials to get the signal profile in percentage signal change (PSC) across 20 s of visual stimulus.

## Results

**Figure 2.**
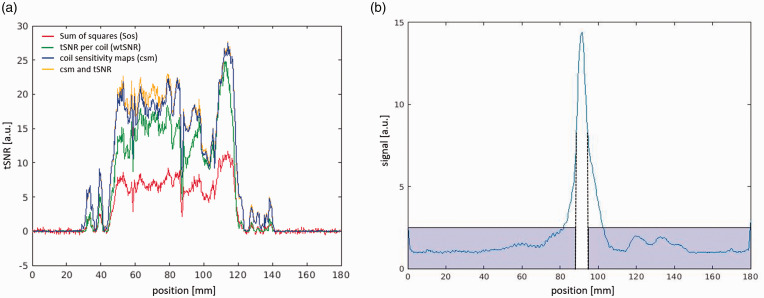
(a) tSNR for different coil combinations for an example dataset: sum of squares (red curve, SoS) and weighted combinations using tSNR per single channel (green curve, tSNR per coil), synthetic coil sensitivity maps (evaluated from data acquired with the phase-encoding enabled and applied saturation pulses for OVS (blue curve, csm) and merged combinations of the previous two methods (yellow curve, csm and tSNR). (b) Signal profile perpendicular to the line for a representative subject, to assess the signal suppression outside the region of interest, OVS slabs are indicated by the purple rectangles. The line-width is measured as the FWHM of the signal profile, represented by the dashed black lines.

Figure 2(a) shows the tSNR along the line-scan direction for the four different coil combinations, for one run of line-scanning data of a representative subject.

All weighted combinations resulted in an increase in tSNR compared to the simple SoS coil combination for reconstruction. Importantly, the weighted combination of both tSNR and csm outperformed the other approaches in terms of tSNR in all individual datasets. In pilot experiments, this result was consistent across volunteers and runs. Hence, this coil combination was used for all subsequent analyses.

Figure 2(b) shows the signal profile perpendicular to the line to assess the signal suppression outside the region of interest. Note that the magnitude values are shown in this plot, hence noise in the suppressed regions is amplified due to the imaging data being complex.

Data are shown for a representative subject. The signal within the line of interest has a peaked profile, i.e. it does not reach a plateau between the two OVS slabs. The OVS slabs are nominally spaced 4 mm apart, hence 16 × 0.25 mm phase-encoded voxels would be contributing signal if the OVS profile were an ideal step-function. Experimentally, on average across all subjects, the mean line width (FWHM) was 6.9 ± 1.0 mm (mean ± std), hence more phase-encoded voxels are effectively contributing to the line-scanning signal.

Table 2 contains the FWHM and OVS values for all subjects, as well as the % of the signal coming from the line and the % signal leakage.

**Table 2.** Percentage of saturated signal outside the line, residual signal inside the line and full-width-at-half maximum of the line profile, signal originating from the line and signal leakage, for all subjects.

Subject	Saturated signal outside line [%]	Residual signal inside line [%]	FWHM [mm]	Signal coming from the line [%]	Signal leakage [%]
1	92.0	83.5	6.7	39.9	60.1
2	94.8	57.8	6.5	37.1	62.9
3	95.0	54.3	7.0	44.7	55.3
4	94.9	55.3	8.3	40.3	59.7
5	94.2	63.2	5.9	48.9	51.1
6	96.3	51.7	5.6	49.2	50.8
7	92.8	75.2	8.5	40.0	60.0
8	94.3	51.1	6.8	36.3	63.7
mean	94.3	61.5	6.9	42.0	58.0
std	1.3	11.8	1.0	5.0	5.0

The performance of the OVS was adequate (see also Figure 1(e)). On average, the suppression of undesired signal outside the region of interest (i.e. where saturations slabs were positioned) was 94.3 ± 1.3% (mean ± std over subjects). This equals to 5.7 ± 1.3% (mean ± std over subjects) of signal that was still present outside the region of interest. The signal within the line of interest is also reduced due to saturation slabs, but the residual signal inside the line is on average 61.5 ± 11.8% (mean ± std over subjects).

Regarding the estimation of signal coming from inside and outside the line, compared to the signal coming from the all the LSD image, we estimated that, on average across subjects, 42 ± 5% (mean ± std over subjects), of the signal is coming from the line, while 58 ± 5% (mean ± std over subjects) is originating from outside the line.

**Figure 3.**
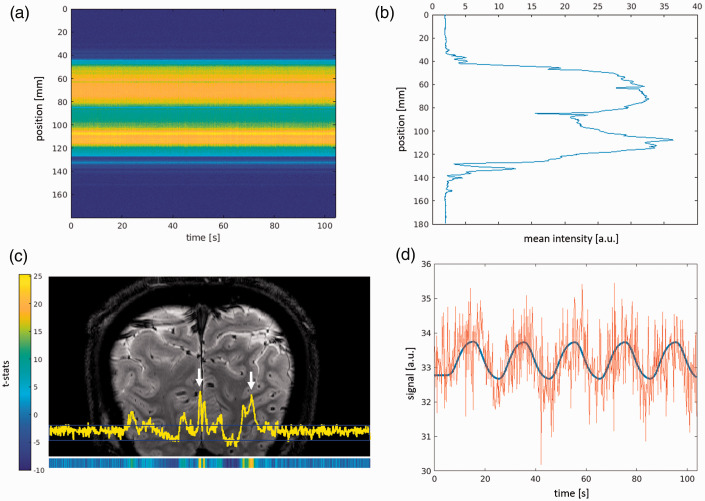
(a) Line-scanning data, averaged over 6 runs and (b) mean intensity signal over the line for a representative subject. (c) t-stats (plot in yellow and colormap on the bottom) superimposed on the anatomical scan for the acquired slice. In the color map, yellow colors represent the highest t-stats and dark blue colors negative t-stats. The position of the line is indicated with a blue box. White arrows indicate voxels with highest activity. (d) time course for a voxel with the raw time-series in orange and the predicted BOLD responses from the GLM in blue.

An example line-scanning dataset of a representative subject, averaged over 6 runs, is shown in Figure 3(a). The color map represents the signal intensity as a function of position (vertical axis) and time (horizontal axis). The stability over time and the limited effect of subject motion in the left-right direction is clear from the stability of the horizontal bands of signal in Figure 3(a). The motion in the left-right direction, estimated for every run and subject through the root mean square displacement of the line center of mass, was on average 0.32 ± 0.14 mm (mean ± std over runs and subjects), apart from one subject which was rejected from the analysis, since the averaged root mean square displacement across runs was 0.85 ± 0.22 mm (mean ± std over runs). Subject motion estimated from LSD images acquired before and after each run showed that the average displacement over the whole scan session (around 40 minutes) was 0.71 ± 0.65 mm (mean ± std over subjects). Figure 3(b) shows the mean signal intensity profile along this line through the occipital lobe. This mean signal also represents the anatomical profile along the line.

Figure 3(c) shows t-stats overlaid on the anatomical scan for a representative subject. The white arrows indicate the voxels with highest activation. Note that there is good spatial correspondence between the positive BOLD t-stats and the grey matter ribbon in the depicted dataset. In Figure 3(d), an example time course for a single voxel (t-stat = 25) is plotted, showing a strong BOLD response along with the predicted BOLD response from the GLM in blue.

**Figure 4.**
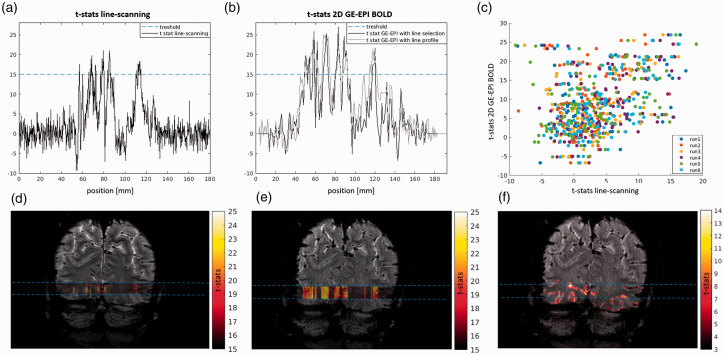
(a) t-stats profile for the line-scanning BOLD data (mean over 6 runs). Blue dashed lines indicate the thresholds (here t-stats that are greater than 15) for showing active voxels in (d) coronal slice (anatomical reference) shown with phase-encoding and without OVS applied. Overlaid on the slice are t-stats of the line scanning data plotted in hot colors (red to yellow). Light blue lines highlight the position of the line-scanning data. (b) t-stats profile for the GE-EPI BOLD data obtained in the two ways: GLM approach considering the line profile (multiplication of LSD prior to functional analysis, gray) and mean signal over the nominal line of interest (black). (e) t-stats obtained with line profile, overlaid on the GE-EPI. Light blue lines highlight the position of the line-scanning data. (c) correlation between t-stats of line-scanning acquisition and 2D GE-EPI with line profile approach for all runs, for the same representative subject. (f) 2 D t-stats for the GE EPI acquisition, showing that most of the activation is coming from the region inside the line, marked with light blue lines.

Figure 4 shows the comparison of line-scanning (4a) and single-slice, TR-matched, GE-EPI BOLD fMRI (4 b). To compare the single-slice GE-EPI and line-scanning data along the line, the GE-EPI scan was multiplied by the LSD image, in order to obtain a version of GE-EPI with the same line profile as the line-scanning dataset. The signal was then summed in the phase-encoding direction in order to obtain the activation profile for the GE-EPI data (grey lines). Using a perfect step function to select the line signal in the GE-EPI (black line, Figure 4(b)), a similar activation pattern is found.

Figure 4(c) shows a scatter plot of the t-stats of the single-slice GE-EPI and line-scanning BOLD fMRI data, resampled to match the spatial resolution of the GE-EPI scan. Data is shown for all runs of a representative subject.

Figure 4(d) shows the GLM results over the whole slice for the GE-EPI acquisition, demonstrating that the bulk of activation in the slice is located inside the line of interest.

The similarity of line-scanning t-stats and GE-EPI BOLD t-stats obtained with the line profile (CASE1-LSD) is quantified for all subjects in Table S1 of the supplementary material, through the correlation between the t-stats for the two scans. The mean value of correlation over all runs and subjects was R_CASE1-LSD_ = 0.59 ± 0.17 (mean ± std).

Similar results were obtained with the second approach of comparison (CASE2-NOM), selection of the nominal line location in the GE-EPI prior to GLM analysis. In this case the mean correlation coefficient across runs and subjects was R_CASE2-NOM_ = 0.49 ± 0.21 (mean ± std, see Table S2 in the supplementary material, and Figure S2 for the t-stats comparison of the same representative subject of Figure 4. The correlation coefficients of the t-stats sampled from the GE-EPI in the two approaches were R_CASE1,2_= 0.81 ± 0.12 (mean ± std) on average over subjects, indicating very little differences between the two approaches (see Figure S3).

Further analysis has been reported in the supplementary material, where Figure S4 shows the power spectra of the line-scanning sequence (Figure S4(a)) and the adapted GE-EPI to have one dimensional data using the line profile, i.e. multiplication of LSD image (Figure S4(b)). The time-series data was first averaged over all voxels in the line contains data before computing the power spectrum. In both cases the physiological contributions from the heart rate and respiration are visible.

To evaluate the stability over time of the six different line-scanning runs, we report in Figure S5 the correlation between t-stats of line-scanning runs (Spearman’s correlation coefficient, R), averaged across subjects. Averaging across all runs and subjects we find that the mean correlation coefficient is R = 0.77 ± 0.04 (mean ± std), hence t-stats are stable over time across the 6 different runs, facilitating averaging over runs.

**Figure 5.**
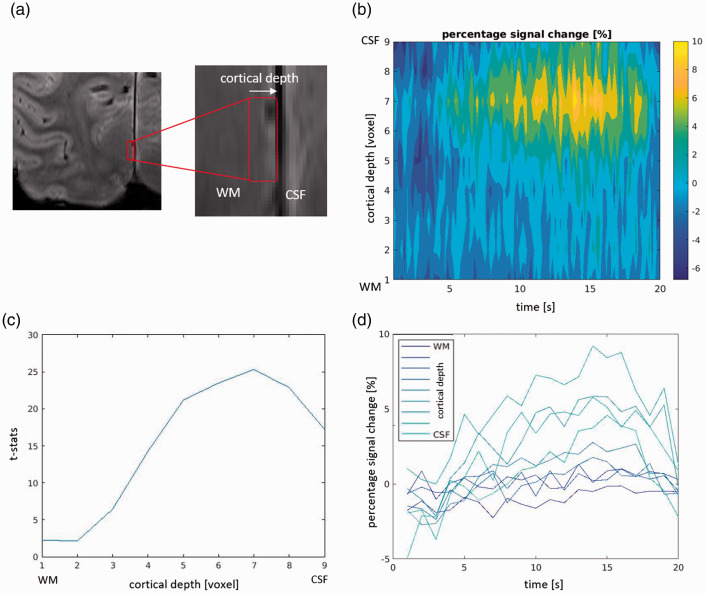
Line-scanning cortical depth analysis; (a) the region of interest (ROI) is depicted by the red box on top of the anatomical image. (b) The BOLD response amplitude (PSC) across cortical depth and time is shown for the ROI. (c) t-stats values for the same voxels. Highest t-stats are found for voxels containing gray matter. (d) mean PSC every 5 timepoints versus time, showing different behaviour in time, depending on cortical depth. PSC stands for percentage signal change.

In order to show the potential of line-scanning for assessment across cortical layers, we show in Figure 5 an example of a layer dependent profile for a small portion of visual cortex located on the edge of the calcarine sulcus, intersected perpendicularly by the line. In Figure 5(a), the region of interest is highlighted through the red box and the percentage signal change is plotted for the same region across time (Figure 5(b)). Figure 5(c) shows the t-statistic values for the same portion of brain, while in Figure 5(d) the PSC versus time is plotted for every voxel separately.

## Discussion

In this paper, we report line-scanning fMRI results in humans combining very high spatial and temporal resolution. Line-scanning has unique potential for laminar fMRI due to its ability to map the BOLD signal response at the mesoscopic scale in humans, with a temporal resolution of a few hundred milliseconds. We demonstrate that line-scanning can detect BOLD activation in human visual cortex, with similar results as standard single-slice GE-EPI, but with a much higher spatial resolution.

We first focused on the optimization of coil combinations for the reconstruction of line-scanning data. A coil combination including coil sensitivity maps and coil channel tSNR for the reconstruction shows the best temporal stability, with resulting tSNR values that are comparable to sub-milllimeter 3D imaging and sufficient for BOLD signal detection.^25^

The line-scanning method demonstrated here relies on the use of saturation pulses that aim to suppress the signal outside the line of interest. The saturation pulses resulted in a good OVS but also reduced the signal inside the line. Considering the signal coming from the line only, with respect to the total signal of the whole LSD image, we estimate that there is, on average, 58% of signal coming from outside the line. For future studies we will investigate whether the rotation of the slice would reduce signal leakage from outside the line, as we could diminish the amount of tissue orthogonal to the line. Future line-scanning efforts could also benefit from sharper saturation profiles or spin-echo based line-scanning (i.e. beam excitation using orthogonal 90° and 180° selective RF pulses^41^ to improve the sharpness of the line profile. At present, however, we argue that the strong correlations of t-stats between dimension-adjusted planar (2 D-EPI) obtained through CASE1-LSD and CASE2-NOM (R = 0.81 ± 0.12) indicate that line-scanning can be used for fMRI with very limited within slice, out-of-line BOLD signal contributions. As quantification of the out-of-line BOLD signal contribution we calculated the difference between R^2^_CASE1-LSD_ and R^2^_CASE2-NOM_, R^2^_CASE1-LSD_-R^2^_CASE2-NOM_= 10.8%, which indicates a variance mismatch of 10.8% between the actual obtained LS activation profile CASE1-LSD and the ideal CASE2-NOM.

Motion correction of fMRI data usually requires spatial information from the images themselves. In line-scanning data, these spatial references are severely reduced when phase-encoding gradients are removed. For this reason, subjects’ movements have to be limited. To prevent motion, subjects were secured in place using foam pads between the ears and the head coil. Moreover, movement in left-right direction is directly visible in the line-scanning data as an instability of the horizontal bands of signal (cfr Figure 3(a)), as well as quantifiable by the displacement of the line center of mass. In these datasets, data from one subject were rejected due to excessive movement. As described in the methods section, a phase-encoded scan containing a 2D image with OVS slabs was repeated before and after the functional scans, to evaluate the subject’s total displacement. All subjects showed little displacement (mean framewise displacement 0.71 ± 0.65 mm, over the 40 minute scan session), apart from the aforementioned rejected dataset. Future studies may investigate subjects’ motion through a multi-slice acquisition repeated multiple times during the scan session, in order to better quantify the exact location of the imaged line in 3D – perhaps in conjunction with a bite-bar to minimize head motion. Moreover multi-echo acquisitions can be used to retrospectively remove physiological noise contributions.^42^ Finally, prospective motion correction through external cameras or fat-navigators could be introduced to correct the position of the line during line-scanning acquisition, without increasing the number of scans needed in our protocol.^43^

In this study, we examined line-scanning sensitivity to BOLD activity using a simple block design visual task. BOLD responses can be detected in the human primary visual cortex, with similar sensitivity to a matched GE-EPI BOLD acquisition and the potential of extraordinary temporal and spatial resolution along the line of interest. The parameters of the two scans were matched as much as possible and the only difference was the FA (16° vs 30°). The difference in FA will result in a 1.1% difference for the inherent SNR between the two scans. This can lead to small differences in the BOLD contrast weighting and image contrast. Differences in flip angles will also lead to different sensitivity to the inflow effect. However, from literature^44,45^ we know that at ultra- high field strengths the inflow effect for BOLD imaging is already minor because of the largely diminished intravascular contribution due to the shortened blood T2*. Previous work by Gao et al.^44^ compared BOLD signals with different flip angles (30°, 60° and 90°) at 3 T. They found that even with such large differences in flip angles, the BOLD percentage signal change is only slightly affected by different in-flow effects. For these reasons, we believe that in our acquisitions a difference of only 14° in flip angle should not affect the interpretation of our results.

The comparison of single-slice GE-EPI BOLD and functional line-scanning ensured that the proposed technique offers similar BOLD sensitivity to conventional approaches. Note that for the current conservative data analysis no temporal filtering was applied, meaning that the raw signal shows the robustness of the proposed approach. Effective signal to noise ratios may also be improved by examining and removing contributions of physiological and movement-related noise sources, a strategy facilitated by the very high sampling rates of our line scanning approach. Moreover, the stability of signals across different runs guarantee that our method is stable over time and can be used for longer tasks. This opens up the possibility of using line-scanning for cognitive neuroscience experiments. Here, the promise is that the spatial and temporal specificities of line-scanning will allow us to investigate time-resolved cognitive computations with laminar precision. In this paper, we describe a line-scanning implementation and assessed its reliability in detecting the BOLD signal changes upon a visual stimulus. Line-scanning was primarily developed for layer fMRI investigations, for which we presented a preliminary example of cortical depth profile where the line was positioned perpendicular to the cortical ribbon in the calcarine sulcus. The cortical depth profile shown here is affected by additional blurring not related to the finite readout gradients, which was estimated to be on the order of 3% (for a T2* value of 25 ms for gray matter), i.e. ∼8µm. Future work could focus on the unexplained blurring which we attribute to biological point-spread function.^46^

Line-scanning fMRI is a promising technique for neuroscience and (patho)physiological research on cerebrovascular and related disorders. Line-scanning fMRI capitalizes on the high spatial and temporal information from BOLD responses across the cortical depth that can yield important insight on microvessel function in health and disease. More specifically, a wide range of neuroscientific questions may be addressed by studying the dynamics of the BOLD response across cortical depth, for example, in integration of visual information across the “blind spot” ;^47^ the dynamics of BOLD responses in higher order areas compared to lower input ones (e.g. output of V1 becoming input in V2 or MT) or the timing of signals in the somatosensory cortex on self-touch, that prevent ticklishness. Regarding elucidating cerebrovascular (patho)physiology, the benefits of line scanning fMRI can be very valuable in identifying and separating microvessels signal features from large vessel signals. Impaired microvessel function directly feeding the neurons as opposed to those draining from the neurons may have very different implications on the nature and origin of cerebrovascular and neurodegenerative diseases.^48–51^ Commonly obtained BOLD signals are usually a mixture from the tissue-feeding microvessels, directly part of the neurovascular network engaged in brain tissue functioning, and signals from the larger venous vessels that drain the cortical tissue. Unfortunately, signals from larger vessels generally obscure signals from the microvasculature, hampering the identification of impaired microvessel function. Nascent high-resolution techniques such as line-scanning will open new methodological avenues to isolate and characterize microvessel spatiotemporal behaviour, i.e. acting as a hemodynamic probe. For example, microvascular flow patterns and transit time estimates in response to neuronal or vascular (i.e. hypercapnia, hyperoxia) challenges could be studied in relation to, for example, small vessel diseases,^52^ and the proposed capillary transtit time heterogeneity model.^53–56^ Finally, characterizing the microvascular hemodynamics by line-scanning could potentially provide more insights in neurovascular coupling and supply input to computational BOLD models.^57,58^

## Conclusion

Overall, we demonstrate the feasibility of line-scanning in humans at 7 T. We show reliable BOLD responses at sub-millimeter and sub-second resolution using the line-scanning fMRI technique, revealing high spatial specificity for a visual task. We demonstrate the robustness of the line-scanning technique by the comparison with a standard method (2D GE-EPI).

## Supplemental Material

sj-pdf-1-jcb-10.1177_0271678X211037266 - Supplemental material for A line through the brain: implementation of human line-scanning at 7T for ultra-high spatiotemporal resolution fMRI Click here for additional data file. Supplemental material, sj-pdf-1-jcb-10.1177_0271678X211037266 for A line through the brain: implementation of human line-scanning at 7T for ultra-high spatiotemporal resolution fMRI by Luisa Raimondo, Tomas Knapen, ĺcaro A.F Oliveira, Xin Yu, Serge O Dumoulin, Wietske van der Zwaag and Jeroen C.W Siero in Journal of Cerebral Blood Flow & Metabolism

## References

[1] Ogawa S Lee TM Kay AR , et al. Brain magnetic resonance imaging with contrast dependent on blood oxygenation. Proc Natl Acad Sci USA 1990; 87: 9868–9872. 2124706 10.1073/pnas.87.24.9868 PMC55275

[2] Dumoulin SO Fracasso A van der Zwaag W , et al. Ultra-high field MRI: advancing systems neuroscience towards mesoscopic human brain function. NeuroImage 2018; 168: 345–357. 28093360 10.1016/j.neuroimage.2017.01.028

[3] Siero JCW Ramsey NF Hoogduin H , et al. BOLD specificity and dynamics evaluated in humans at 7 T: comparing gradient-echo and spin-echo hemodynamic responses. PLoS ONE 2013; 8: e54560–e54568. 23336008 10.1371/journal.pone.0054560 PMC3546000

[4] Narsude M Gallichan D van der Zwaag W , et al. Three-dimensional echo planar imaging with controlled aliasing: a sequence for high temporal resolution functional MRI. Magn Reson Med 2016; 75: 2350–2361. 26173572 10.1002/mrm.25835

[5] Duyn JH. The future of ultra-high field MRI and fMRI for study of the human brain. NeuroImage 2012; 62: 1241–1248. 22063093 10.1016/j.neuroimage.2011.10.065 PMC3389184

[6] Pohmann R Speck O Scheffler K. Signal-to-noise ratio and MR tissue parameters in human brain imaging at 3, 7, and 9.4 tesla using current receive coil arrays. Magn Reson Med 2016; 75: 801–809. 25820458 10.1002/mrm.25677

[7] van der Zwaag W Francis S Head K , et al. fMRI at 1.5, 3 and 7 T: characterising BOLD signal changes. NeuroImage 2009; 47: 1425–1434. 19446641 10.1016/j.neuroimage.2009.05.015

[8] Yacoub E van de Moortele PF Shmuel A , et al. Signal and noise characteristics of Hahn SE and GE BOLD fMRI at 7 T in humans. NeuroImage 2005; 24: 738–750. 15652309 10.1016/j.neuroimage.2004.09.002

[9] Fischl B Dale AM. Measuring the thickness of the human cerebral cortex from magnetic resonance images. Proc Natl Acad Sci USA 2000; 97: 11050–11055. 10984517 10.1073/pnas.200033797 PMC27146

[10] Petridou N Siero JCW. Laminar fMRI: What can the time domain tell us? NeuroImage 2019; 197: 761–771. 28736308 10.1016/j.neuroimage.2017.07.040 PMC5775945

[11] Goense JBM Logothetis NK. Laminar specificity in monkey V1 using high-resolution SE-fMRI. Magn Reson Imaging 2006; 24: 381–392. 16677944 10.1016/j.mri.2005.12.032

[12] Lee J Ehlers C Crews F , et al. Automatic cortical thickness analysis on rodent brain. Proc SPIE Int Soc Opt Eng 2011; 7962: 7962481–79624811. 21909228 10.1117/12.878305 PMC3168533

[13] Hirano Y Stefanovic B Silva AC. Spatiotemporal evolution of the functional magnetic resonance imaging response to ultrashort stimuli. J Neurosci 2011; 31: 1440–1447. 21273428 10.1523/JNEUROSCI.3986-10.2011 PMC3078723

[14] Poplawsky AJ Fukuda M Murphy M , et al. Layer-specific fMRI responses to excitatory and inhibitory neuronal activities in the olfactory bulb. J Neurosci 2015; 35: 15263–15275. 26586815 10.1523/JNEUROSCI.1015-15.2015 PMC4649002

[15] Silva AC Koretsky AP. Laminar specificity of functional MRI onset times during somatosensory stimulation in rat. Proc Natl Acad Sci U S A 2002; 99: 15182–15187. 12407177 10.1073/pnas.222561899 PMC137564

[16] Koopmans PJ Barth M Norris DG. Layer-specific BOLD activation in human V1. Hum Brain Mapp 2010; 31: 1297–1304. 20082333 10.1002/hbm.20936 PMC6870878

[17] Siero JCW Hendrikse J Hoogduin H , et al. Cortical depth dependence of the BOLD initial dip and poststimulus undershoot in human visual cortex at 7 tesla. Magn Reson Med 2015; 73: 2283–2295. 24989338 10.1002/mrm.25349 PMC4282631

[18] Polimeni JR Fischl B Greve DN , et al. Laminar analysis of 7T BOLD using an imposed spatial activation pattern in human V1. NeuroImage 2010; 52: 1334–1346. 20460157 10.1016/j.neuroimage.2010.05.005 PMC3130346

[19] Jesmanowicz A Bandettini PA Hyde JS. Single-shot half k-space high-resolution gradient-recalled EPI for fMRI at 3 tesla. Magn Reson Med 1998; 40: 754–762. 9797160 10.1002/mrm.1910400517

[20] Pruessmann KP Weiger M Scheidegger MB , et al. SENSE: sensitivity encoding for fast MRI. Magn Reson Med 1999; 42: 952–962. 10542355

[21] Griswold MA Jakob PM Heidemann RM , et al. Generalized autocalibrating partially parallel acquisitions (GRAPPA). Magn Reson Med 2002; 47: 1202–1210. 12111967 10.1002/mrm.10171

[22] Geethanath S Reddy R Konar AS , et al. Compressed sensing MRI: a review. Crit Rev Biomed Eng 2013; 41: 183–204. 24579643 10.1615/critrevbiomedeng.2014008058

[23] Setsompop K Feinberg DA Polimeni JR. Rapid brain MRI acquisition techniques at ultra-high fields. NMR Biomed 2016; 29: 1198–1221. 26835884 10.1002/nbm.3478 PMC5245168

[24] Poser BA Koopmans PJ Witzel T , et al. Three dimensional echo-planar imaging at 7 tesla. NeuroImage 2010; 51: 261–266. 20139009 10.1016/j.neuroimage.2010.01.108 PMC2853246

[25] van der Zwaag W Marques JP Kober T , et al. Temporal SNR characteristics in segmented 3D-EPI at 7T. Magn Reson Med 2012; 67: 344–352. 21656557 10.1002/mrm.23007 PMC3627735

[26] Moeller S Yacoub E Olman CA , et al. Multiband multislice GE-EPI at 7 tesla, with 16-fold acceleration using partial parallel imaging with application to high spatial and temporal whole-brain FMRI. Magn Reson Med 2010; 63: 1144–1153. 20432285 10.1002/mrm.22361 PMC2906244

[27] Reynaud O Jorge J Gruetter R , et al. Influence of physiological noise on accelerated 2D and 3D resting state functional MRI data at 7 T. Magn Reson Med 2017; 78: 888–896. 28686788 10.1002/mrm.26823

[28] Pfeuffer J van de Moortele PF Yacoub E , et al. Zoomed functional imaging in the human brain at 7 tesla with simultaneous high spatial and high temporal resolution. NeuroImage 2002; 17: 272–286. 12482083 10.1006/nimg.2002.1103

[29] Duong TQ Yacoub E Adriany G , et al. High-resolution, spin-echo BOLD, and CBF fMRI at 4 and 7 T. Magn Reson Med 2002; 48: 589–593. 12353274 10.1002/mrm.10252

[30] Cheng K Waggoner RA Tanaka K. Cheng01. Neuron 2001; 32: 359–316. 10.1016/s0896-6273(01)00477-9 11684004

[31] Yacoub E Harel N Ugurbil K. High-field fMRI unveils orientation columns in humans. Proc Natl Acad Sci USA 2008; 105: 10607–10612. 18641121 10.1073/pnas.0804110105 PMC2492463

[32] Huber L Handwerker DA Jangraw DC , et al. High-Resolution CBV-fMRI allows mapping of laminar activity and connectivity of cortical input and output in human M1. Neuron 2017; 96: 1253–1263.e7. 29224727 10.1016/j.neuron.2017.11.005 PMC5739950

[33] Kashyap S Ivanov D Havlicek M , et al. Resolving laminar activation in human V1 using ultra-high spatial resolution fMRI at 7T. Sci Rep 2018; 8: 17063. 30459391 10.1038/s41598-018-35333-3 PMC6244001

[34] Yu X Qian C Chen DY , et al. Deciphering laminar-specific neural inputs with line-scanning fMRI. Nat Methods 2014; 11: 55–58. 24240320 10.1038/nmeth.2730 PMC4276040

[35] Spitzer D Bauer J Faber C. Feasibility of line scanning BOLD fMRI on human subjects. In: *ISMRM 24th annual meeting & exhibition proceedings*, Singapore, 30 May–5 June 2016, pp. 16–18. Concord, CA: ISMRM.

[36] Morgan AT Nothnagel N Petro LS , et al. High-resolution line-scanning reveals distinct visual response properties across human cortical layers. *bioRxiv* 2020: 1–17.

[37] Siero JCW de Oliveira IAF Choi S , et al. Human line-scanning fMRI: initial results of ultra-high temporal and spatial resolution hemodynamic imaging. J Cereb Blood Flow Metab 2019; 39: 1–123.

[38] Raimondo L Knapen T de Oliveira IAF , et al. Preliminary results of functional line-scanning in humans: submillimeter, subsecond resolution evoked responses. Magn Reson Mater Phy 2019. 32: 235–371.

[39] Power JD Barnes KA Snyder AZ , et al. Spurious but systematic correlations in functional connectivity MRI networks arise from subject motion. NeuroImage 2012; 59: 2142–2154. 22019881 10.1016/j.neuroimage.2011.10.018 PMC3254728

[40] Roemer PB Edelstein WA Hayes CE , et al. The NMR phased array. Magn Reson Med 1990; 16: 192–225. 2266841 10.1002/mrm.1910160203

[41] Choi S Zeng H Pohmann R , et al. Novel alpha-180 SE based LINE-scanning method (SELINE) for laminar-specific fMRI. In: *ISMRM 27th annual meeting & exhibition proceedings*, Montreal, Canada, 11–16 May 2019, p.1166. Concord, CA: ISMRM.

[42] Kundu P Voon V Balchandani P , et al. Multi-echo fMRI: a review of applications in fMRI denoising and analysis of BOLD signals. NeuroImage 2017; 154: 59–80. 28363836 10.1016/j.neuroimage.2017.03.033

[43] Zaitsev M Akin B LeVan P , et al. Prospective motion correction in functional MRI. Neuroimage 2017; 154: 33–42. 27845256 10.1016/j.neuroimage.2016.11.014 PMC5427003

[44] Gao JH Liu HL. Inflow effects on functional MRI. NeuroImage 2012; 62: 1035–1039. 22019882 10.1016/j.neuroimage.2011.09.088

[45] Gao JH Miller I Lai S , et al. Quantitative assessment of blood inflow effects in functional MRI signals. Magn Reson Med 1996; 36: 314–319. 8843386 10.1002/mrm.1910360219

[46] Fracasso A Dumoulin SO Petridou N. Point-spread function of the BOLD response across columns and cortical depth in human extra-striate cortex. Prog Neurobiol 2021; 202: 102034. 33741401 10.1016/j.pneurobio.2021.102034 PMC8958221

[47] de Hollander G van der Zwaag W Qian C , et al. Ultra-high field fMRI reveals origins of feedforward and feedback activity within laminae of human ocular dominance columns. NeuroImage 2021; 228: 117683. 33385565 10.1016/j.neuroimage.2020.117683

[48] Sweeney MD Kisler K Montagne A , et al. The role of brain vasculature in neurodegenerative disorders. Nat Neurosci 2018; 21: 1318–1331. 30250261 10.1038/s41593-018-0234-x PMC6198802

[49] Iturria-Medina Y Sotero RC Toussaint PJ , et al.; Alzheimer’s Disease Neuroimaging Initiative. Early role of vascular dysregulation on late-onset Alzheimer’s disease based on multifactorial data-driven analysis. Nat Commun 2016; 7: 11934. 27327500 10.1038/ncomms11934 PMC4919512

[50] Pantoni L. Cerebral small vessel disease: from pathogenesis and clinical characteristics to therapeutic challenges. Lancet Neurol 2010; 9: 689–701. 20610345 10.1016/S1474-4422(10)70104-6

[51] Nielsen RB Egefjord L Angleys H , et al. Capillary dysfunction is associated with symptom severity and neurodegeneration in Alzheimer’s disease. Alzheimers Dement 2017; 13: 1143–1153. 28343848 10.1016/j.jalz.2017.02.007

[52] Zwanenburg JJM van Osch MJP. Targeting cerebral small vessel disease with MRI. Stroke 2017; 48: 3175–3182. 28970280 10.1161/STROKEAHA.117.016996

[53] Angleys H Østergaard L Jespersen SN. The effects of capillary transit time heterogeneity (CTH) on brain oxygenation. J Cereb Blood Flow Metab 2015; 35: 806–817. 25669911 10.1038/jcbfm.2014.254 PMC4420854

[54] Østergaard L Jespersen SN Mouridsen K , et al. The role of the cerebral capillaries in acute ischemic stroke: the extended penumbra model. J Cereb Blood Flow Metab 2013; 33: 635–648. 23443173 10.1038/jcbfm.2013.18 PMC3652700

[55] Rasmussen PM Jespersen SN Østergaard L. The effects of transit time heterogeneity on brain oxygenation during rest and functional activation. Journal of Perinatology 2015; 35: 432–442. 10.1038/jcbfm.2014.213 PMC4348381 25492112

[56] Gutiérrez-Jiménez E Angleys H Rasmussen PM , et al. The effects of hypercapnia on cortical capillary transit time heterogeneity (CTH) in anesthetized mice. J Cereb Blood Flow Metab 2018; 38: 290–303. 28181842 10.1177/0271678X17692598 PMC5951010

[57] Havlicek M Uludağ K. A dynamical model of the laminar BOLD response. NeuroImage 2020; 204: 116209. 31546051 10.1016/j.neuroimage.2019.116209

[58] Báez-Yánez MG Siero JC Petridou N. A statistical 3D model of the human cortical vasculature to compute the hemodynamic fingerprint of the BOLD fMRI signal. bioRxiv 2020; 31: 1–63.

