# Supplementary material for: A line through the brain: implementation of human line-scanning at 7T for ultra-high spatiotemporal resolution fMRI
Source: J Cereb Blood Flow Metab. 2021 Aug 20;41(11):2831–43. doi: 10.1177/0271678X211037266 (PMC8756483; doi:10.1177/0271678X211037266)
Supplement: sj-pdf-1-jcb-10.1177_0271678X211037266 - Supplemental material for A line through the brain: implementation of human line-scanning at 7T for ultra-high spatiotemporal resolution fMRI [file sj-pdf-1-jcb-10.1177_0271678X211037266.pdf]

Supplementary material

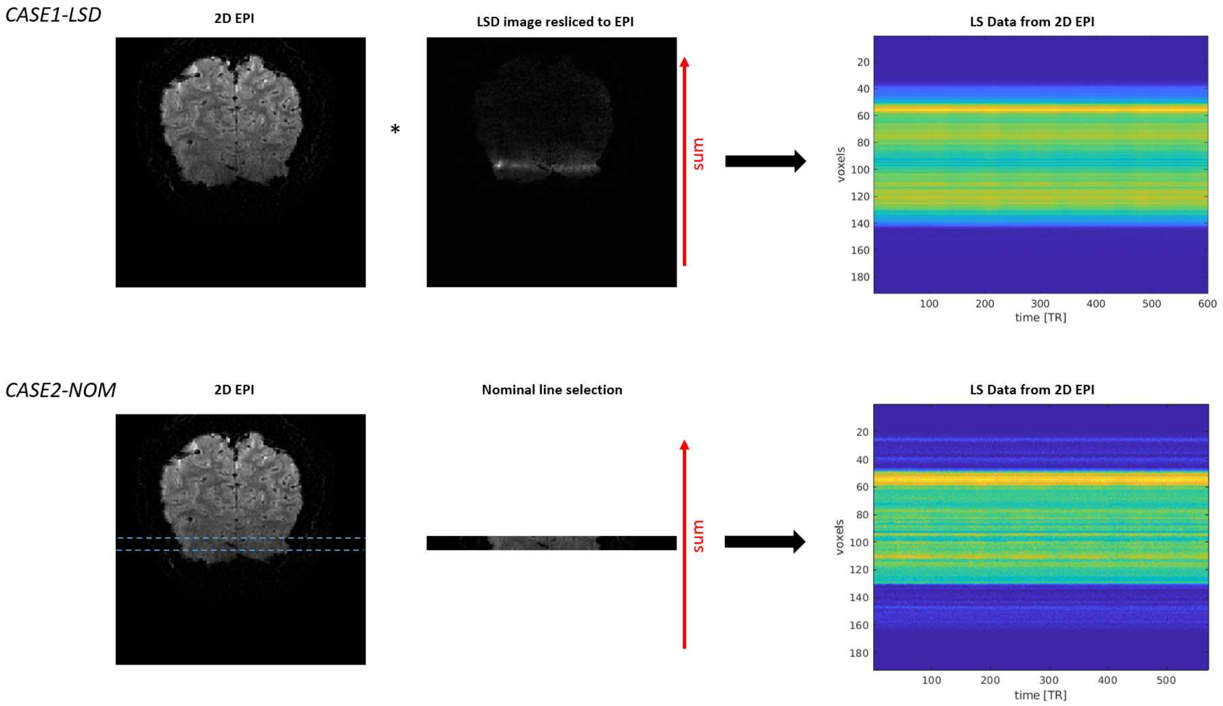

Figure S1: Graphical explanation of CASE1-LSD and CASE2-NOM. **(top row)** 2D EPI multiplied by LSD image, before summing in the phase encoding direction leads to a line-scanning profile for CASE1-LSD. **(bottom row)** nominal selection of the line, before summing in the phase encoding direction and related line-scanning profile for CASE2-NOM.

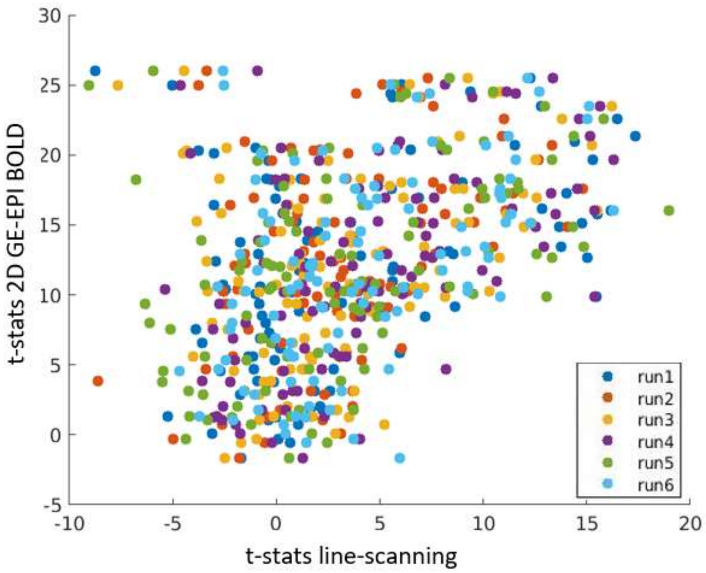

Figure S2: correlation between t stats of line-scanning acquisition and 2D GE-EPI with line selection approach for all runs, for the same representative subject of Figure 8.

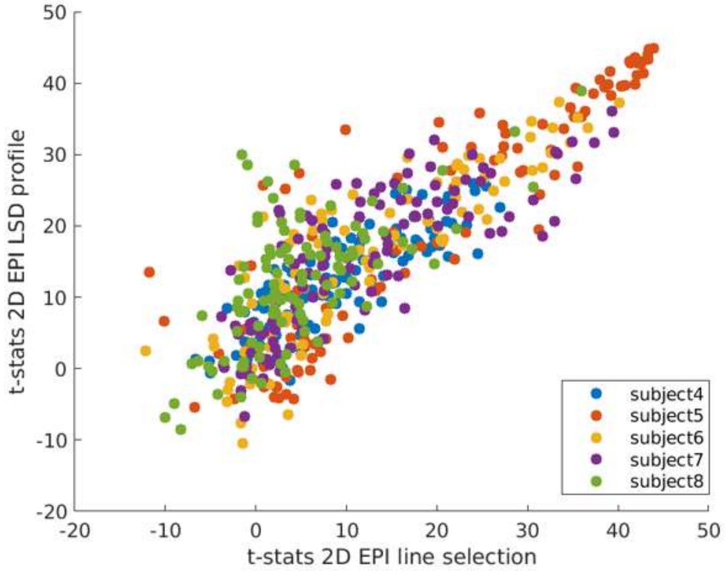

Figure S3: correlation between t stats of 2D GE-EPI evaluated with the two different approaches (line selection and LSD image profile).

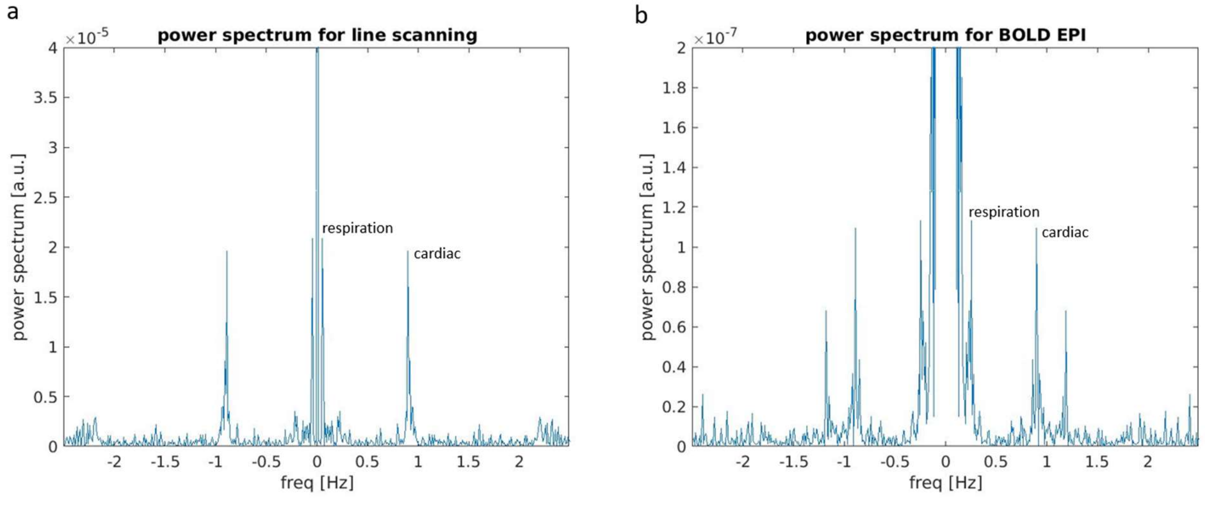

Figure S4: **(a)** power spectrum of line-scanning, and **(b)** power spectrum of the 1D version of GE-EPI obtained through multiplication with the LSD image/ and averaged over all line. For both the line-scanning and GE-EPI power spectra, the timeseries data was first averaged over all voxels in the line before computing the power spectrum.

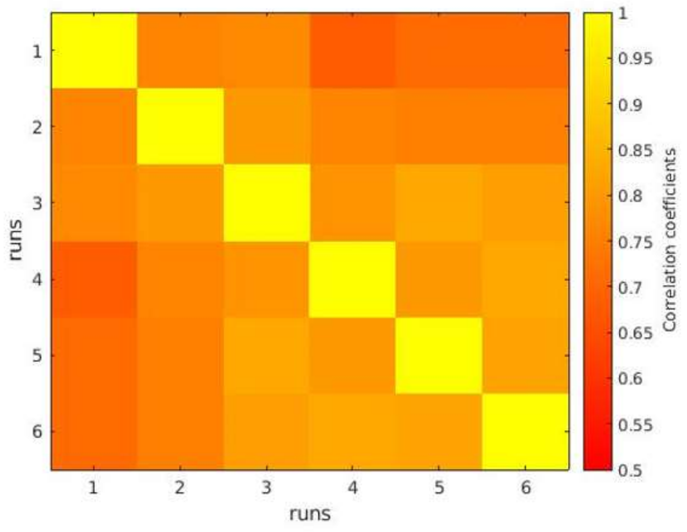

Figure S5: Run-by-run line-scanning t-statistic correlation matrix, averaged across subjects.

Table S1: Correlation between line-scanning and BOLD GE-EPI t-stats evaluated after multiplying the GE-EPI and the LSD image, Spearman correlation coefficients (R) are reported for each run and subject.

subject	4	5	6	7	8
run1	0.38	0.82	0.72	0.54	0.47
run2	0.36	0.86	0.72	0.63	0.43
run3	0.37	0.83	0.74	0.56	0.50
run4	0.52	0.84	0.54	0.52	0.52
run5	0.45	0.83	0.64	0.53	0.43
run6	0.48	0.87	0.69	0.55	0.50
**mean**	0.43	0.84	0.68	0.56	0.48
**std**	0.07	0.02	0.08	0.04	0.04

Table S2: Correlation between line-scanning and BOLD GE-EPI t-stats evaluated after manual line selection in the GE-EPI, Pearson correlation coefficients (R) are reported for each run and subject.

subject	4	5	6	7	8
run1	0.30	0.80	0.64	0.43	0.46
run2	0.30	0.83	0.65	0.55	0.16
run3	0.30	0.79	0.64	0.53	0.28
run4	0.42	0.79	0.45	0.35	0.19
run5	0.39	0.80	0.56	0.45	0.27
run6	0.43	0.79	0.58	0.41	0.25
**mean**	0.36	0.80	0.59	0.45	0.27
**std**	0.06	0.01	0.08	0.07	0.11
